# Takotsubo Cardiomyopathy and β-Blocker Poisoning: A Case Report

**DOI:** 10.3390/medicina58121777

**Published:** 2022-12-02

**Authors:** Nicoleta-Monica Popa-Fotea, Miruna Mihaela Micheu, Cosmin Mihai, Ruxandra State, Radu Tincu, Alexandru Scafa-Udriste

**Affiliations:** 1 Department 4 Cardio-Thoracic Pathology, University of Medicine and Pharmacy “Carol Davila”, 050474 Bucharest, Romania; 2 Department of Cardiology, Emergency Clinical Hospital, 014461 Bucharest, Romania

**Keywords:** Takotsubo cardiomyopathy, β-blocker, benzodiazepine and digoxin poisoning, epinephrine, catecholamines, emotional stress, cardiotoxicity

## Abstract

β-blocker poisoning is frequently observed because of its primary use for the treatment of cardiovascular diseases. The management of β-blocker toxicity is dependent on the cardiovascular response and the severity of presentation. The present study describes the case of a patient with combined drug intoxication, β-blocker, digoxin, benzodiazepines, acetaminophen and opiates in a suicidal attempt. A 63-year-old female was found somnolent and in a confused state at her residence following intentional poly-drug ingestion. Upon presentation, she was found to be hemodynamically unstable and was thus treated with vasopressors. The toxicological screening performed upon presentation was positive for polydrug ingestion. On day 3, the patient developed chest pain and ST-segment elevation in anterior leads, while transthoracic echocardiographic assessment disclosed a non-dilated left ventricle with moderate dysfunction and akinesia of the apex. Coronary angiogram revealed normal coronary arteries and, subsequently, the diagnosis of Takotsubo cardiomyopathy (TTC) was suspected. Supportive treatment was initiated with favorable evolution and left ventricular ejection fraction normalization. The management of hemodynamic instability with vasopressors should be judiciously administered in the treatment of β-blocker poisoning, in view of the adverse effects on cardiac functions, including stress cardiomyopathy.

## 1. Introduction

Beta-blockers are a frequent cause of unintentional or premeditated poisoning associated with significant morbidity and mortality [1]. Complications following β-blocker overdose are a consequence of the adrenergic blockade, resulting in bradycardia, hypotension and, in severe cases, in profound myocardial depression and cardiogenic shock. Other adverse events may occur because of specific agent properties, such as intrinsic sympathomimetic activity, lipophilicity or membrane-stabilizing activity. Catecholamines, vasopressors and high-dose insulin euglycemic therapy are treatment options associated with a reduced mortality rate, as demonstrated in a recent systematic review [2]. However, the requirement for prolonged catecholamine use may lead to detrimental effects, such as Takotsubo cardiomyopathy (TTC), also known as ‘broken heart syndrome’, although such an event is rare (0.5%) [3] yet potentially fatal. Initially, TTC was considered to be a benign condition [4]; however, it was then proven to be possibly associated with severe complications and mortality similar to those observed in acute coronary syndromes [5]. Numerous types of stressful events, either positive or negative, can trigger the development of the disease, such as the sudden loss of loved ones, disasters, accidents, the use of certain drugs, such as cocaine, amphetamines, or the exogenous administration of catecholamines, public speaking, etc. Of note, patients with psychiatric diseases are more prone to developing TTC, which in such cases is not necessarily induced by antipsychotic treatment [6]. Various mechanisms have been proposed for the pathophysiology of the disease, such as body release of catecholamines in various circumstances (which is the most frequently mentioned cause by reviews and case reports [7,8]), and less commonly, lack of estrogen, inflammation or microvascular disease. TTC differs from other cardiomyopathies because of complete recovery of the systolic function at variable time intervals and the absence of the upturn of left ventricular ejection fraction, excluding TTC; the differential diagnosis from pheochromocytoma is made based on free metanephrine and normetanephrine plasma concentrations in the presence of adrenal or extra-adrenal tumors and from myocarditis with the aid of cardiac magnetic resonance imaging and endomyocardial biopsy with Dallas criteria [9].

The present study describes the case of a 63-year-old female with no cardiovascular disease who developed TTC during her treatment for multidrug overdose that also included β-blocker poisoning.

## 2. Case Report

A previously healthy 63-year-old female was found at her residence in a somnolent and confused state, with multiple empty blister packs of metoprolol (12 g), digoxin (7.5 mg), diazepam, acetaminophen/tramadol and chlorzoxazone (for the last three drugs, the ingested amount could not be precisely established). Voluntary ingestion was made in a suicidal attempt following a long period of prolonged conflict with her son. Although she had been previously diagnosed with depression, she had declined the recommendations of her psychiatrist.

Upon arrival at the Emergency Department of Bucharest Clinical Hospital, the patient was bradycardic (heart rate, 51 bpm; sinus bradycardia) with hemodynamic instability (blood pressure of 90/40 mmHg). She was immediately transferred to the intensive care unit with a Glasgow coma scale of 12. A physical examination revealed sinus bradycardia (65 bpm) and hypotension (78/45 mmHg); she was afebrile, with a respiratory rate of 12 breaths/min and a peripheral oxygen saturation of 98% in room air. The general physical examination was otherwise normal. Blood tests revealed normal levels of potassium and glycemia, with a slight increase in lactate levels (1.9 mmol/L) (Table 1). The increase in lactate is most probably due to the increase in lactate dehydrogenase, an enzyme elevated in response to hemodynamic stress. A 12-lead electrocardiogram (ECG) (Edan SE-1200 EKG Machine; EXPRESS BASIC, https://medisal.ro/electrocardiografe/3909-electrocardiograf-edan-se-1200-express-basic.html; accessed on 20 June 2022) showed sinus rhythm, with rare supraventricular ectopics, mild and diffuse ST-segment depressions and normal corrected QT intervals. Liquid chromatography–mass spectrometry (LC-MS) analysis followed a previously published protocol [10] for the determination of various toxic compounds in plasma, including a sample preparation step: 0.5 mL plasma from the patient was spiked with 0.1 mL internal standard at a concentration of 1 μg/mL (chlorpromazine, haloperidol and prazepam) and then diluted with 2 mL of 0.1 M phosphate buffer (pH 6.0). The resulting mix was extracted with a solid-phase extraction method (Thermo Scientific HyperSep Verify-CX 200 mg mixed-mode cartridges; Thermo Fisher Scientific, Inc., Waltham, MA, USA) prior to injecting into the LC-MS. Liquid chromatography separation was performed with Thermo Scientific Vanquish Flex Binary using a Thermo Scientific Accucore Phenyl Hexyl column (100 × 2.1 mm; 5 μm particles; Thermo Fisher Scientific, Inc.) at a flow rate of 200 μL/min and an injection volume of 10 μL. Mass spectrometry was performed on a triple quadrupole mass spectrometer (Thermo Scientific TSQ Quantis Plus; Thermo Fisher Scientific, Inc.) with a negative/positive switching scan-dependent experiment. Data acquisition and processing was performed with Thermo Scientific TraceFinder 5.1 software (Thermo Fisher Scientific, Inc.). The analysis from plasmatic probes harvested at ~1 h following ingestion was positive for poisoning with benzodiazepines (1800 ng/mL), digoxin (4.8 ng/mL) and metoprolol (1 mg/L), but within normal ranges for acetaminophen, opioids and barbiturates (Table 1). Ethanol was not detected in serum and the concentration of digoxin at presentation and at 6 h apart did not exceed 5 ng/mL. As the plasma levels of digoxin were low, specific antibodies were not prescribed to the patient.

The patient was initially challenged with isotonic saline solution (800 mL); however, due to maintained hypotension, she required a continuous intravenous infusion of epinephrine via a central venous catheter at doses between 0.05–0.25 µg/kg/min, as recommended by the 2021 European Society of Cardiology guidelines [11] to maintain a mean arterial pressure of 65 mmHg, at a total dose of 5 mg. As the ingestion was recent (~30 min prior to her arrival at the emergency department), gastric lavage was performed, along with a 1.5 mL/kg 20% lipid bolus followed by a 0.25 mL/kg/min infusion for ~8 h.

**Figure 1:**
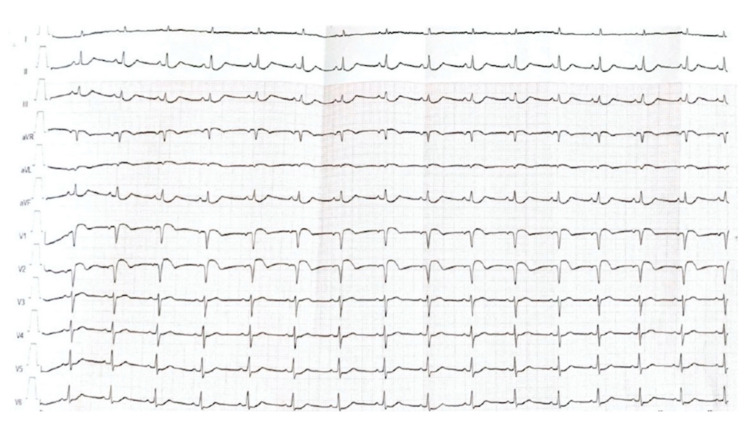
Electrocardiogram depicting sinus rhythm, ST segment elevation in V1 and V2 and mirror image in the inferior leads (DII, DIII and aVF).

The patient was stabilized 4 h later (blood pressure, 120/80 mmHg; sinus rhythm, 80 bpm); however, she was continuously supervised in the intensive care unit. On day 3, 12 h after the interruption of the continuous epinephrine administration, the patient developed intense retrosternal chest pain, with dynamic ECG changes consisting of ST segment elevation in V1 and V2 and mirror image in the inferior leads (Figure 1).

**Figure 2:**
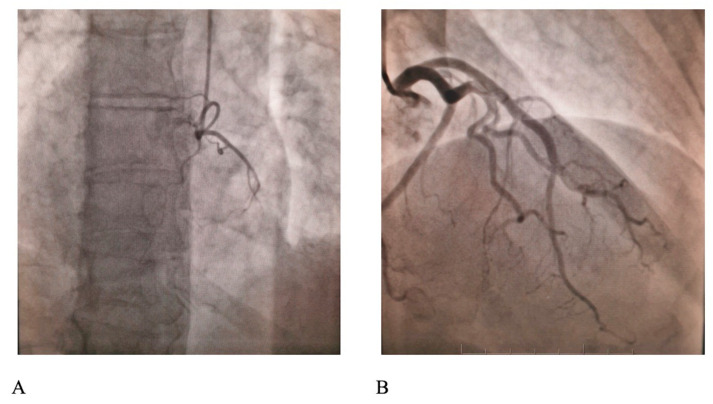
Coronary angiogram displaying normal (**A**) right and (**B**) left coronary arteries.

The troponin I level was 3.4 ng/mL initially and reached peak levels after 3 h (10.8 ng/mL). Transthoracic echocardiographic assessment revealed a non-dilated left ventricle with a moderate reduction in the left ventricle ejection fraction (LVEF, 40%) with severe hypokinesis of the apical segments of the anterior and inferior wall. On the same day, on day 3 from the time of admission, the patient underwent a coronary angiogram using the radial approach with a Glidesheath Slender 6 French (F; cat. no. RM*ES6J10SQ, Terumo Europe NV) using iopamidol contrast (Isovue, Bracco Diagnostics Inc.). The engagement of the right and left coronary arteries was achieved with diagnostic 6 F catheters, Judkins left 3.5 (cat. no. RQ-4JL3500M; Terumo Europe NV) for the left coronary artery and Judkins right 4.0 (RQ-4JR4000M, Terumo Europe NV) for the right artery, on a Siemens Artis zee angiography system (Siemens). The acquisition of at least three incidences for the right coronary artery and at least four for the left coronary artery did not reveal any notable lesions at the level of the epicardial arteries (Figure 2).

The diagnosis of stress cardiomyopathy or TTC was established based on the Mayo Clinic criteria [12] and also by considering earlier expert consensus, namely the 2018 International Takotsubo Diagnostic Criteria [13,14], this being one of the entities included under the umbrella of myocardial infarction with non-obstructive coronary arteries (MINOCA). Various differential diagnosis from the MINOCA, seen as Pandora’s box, were considered in the case of our patient, such as myocarditis, type 2 myocardial infarction or other cardiomyopathies. The lack of cardiac magnetic resonance modifications suggestive for myocardial infarction or Lake Louis myocarditis criteria sustained TTC diagnosis. Supportive treatment with fluid resuscitation was administered (250 mL isotonic saline solution for 15 min, aspirin 75 mg daily, statins (atorvastatin at 80 mg once per day) and continuous noradrenaline infusion at an average dose of 0.5 µg/kg/min for ~12 h followed by progressive dose reduction until discontinuation on the following day); the previously mentioned treatment allowed for hemodynamic improvement on day 6 after drug poisoning. Echocardiographic assessment on day 6 revealed complete recovery of the LVEF with normal wall motion and the patient was discharged on day 8. At the 3-month follow-up, the patient remained asymptomatic whilst being evaluated twice a month in the psychiatric out-patient clinic for a depressive disorder requiring fluoxetine 20 mg daily and cognitive behavioral therapy twice a week.

## 3. Discussion

TTC is an entity whose pathophysiological mechanisms are incompletely understood despite comprehensive research [15]. The high elevation of plasma levels of catecholamines have a negative inotropic effect via the gastrointestinal tract, resulting in the decreased contractility of the left ventricle. This mechanism is very appealing as it may explain the most frequent form of TΤC, the apical one, considering that β-2 receptors are most abundant at the apical level; notwithstanding, there are also other forms of ΤCC that are not yet fully understood with regards to the aforementioned theory. The possible involved pathway may be the increased activity of phosphatidyl inositol 3-kinase-protein kinase B (PI3K/AKT) with a crucial anti-apoptotic role, contributing to rapid recovery in TTC. Epinephrine or other catecholamines have been previously described as causative agents in TΤC [16]; a previous systematic review with a total of 41 cases revealed rapid reversible cardiac dysfunction, although with a good prognosis [17]. These previously described TΤC cases occurred at the recommended dosages of epinephrine and, in some cases, even at a lower-than-usual dose. In a previous study using a mouse model of TTC, it was revealed that epinephrine activated the α-1 receptor and, furthermore, the nicotinamide adenine dinucleotide phosphate-reactive oxygen species-protein kinase C signaling pathway [18]. Several pharmacological substances apart from catecholamines and β-blockers have been found to be involved in TΤC, such as chloroquine, olanzapine, inhibitors of catecholamine reuptake, alcohol, opioid withdrawal and chemotherapy [16]. Another plausible pathophysiological mechanism is the reduction in estrogen levels in post-menopausal women, depriving them from the hormonal protective cardiovascular effects. Estrogens play a role in the downregulation of β-adrenergic receptors and attenuate the stress induced by the hypothalamo-sympatho-adrenal axis from the central nervous system to the heart, consequently leading to a predisposition to the negative effects of excess amounts of catecholamines. Inflammation is also involved in the development of TΤC, even though both the Mayo Clinic criteria [11] and European Society of Cardiology-Heart Failure Association guidelines [12] included a mandatory exclusion of myocarditis. It has been demonstrated that cardiac magnetic resonance imaging reveals late gadolinium enhancement in almost 10% of subjects with TΤC, as well as the enhancement of patchy or focal areas different from myocardial infarction or myocarditis, aiding in the differential diagnosis [19]. The intimate mechanisms through which inflammation induces ventricular dysfunction in TTC are limited, although an increase in the levels of reactive oxygen species is suggested, leading to the injury of myocardial cells, mitochondrial malfunction, a calcium overload, and the activation of in the toll-like receptor 4 (TLR4/NF-κB) pathway with high apoptosis [20]. Microvascular dysfunction is another mechanism involved in ΤCC through the aid of intravascular catheter-based techniques; the index of microvascular resistance measured is extremely increased in the acute phase of TΤC, indicating microvascular dysfunction; however, the extent of its involvement is debatable as this is calculated during the acute event [21]. Although the involvement of catecholamines in the development of cardiomyopathy in the present study was suggested, the level of catecholamines was not determined—measurements that would have provided further insight into the pathophysiology of TTC. However, one study [22] only revealed a moderate elevation; in the case presented herein, perhaps, there were only slightly higher concentrations because of both exogenous and endogenous increments.

Compared with a systematic review [7], where there was a marked clinical presentation with extensive left ventricular dysfunction and a high complication rate, the patient in the present study exhibited only mild LVEF deterioration and no other complications, at least on the middle-term follow-up. This difference may be due to the fact that the majority of the notable cases in the mentioned review were of pheochromocytoma- and paragangliomas-induced TTC rather than by exogenous catecholamines, which is in some contradiction with the European [11] and American guidelines [13] that need the exclusion of these entities for the diagnosis of stress cardiomyopathy.

As highlighted in another review [23], the better stratification of the risk and long-term prognosis of TTC cases is a requisite as a number of subjects present with recurrent major adverse cardiac and cerebrovascular events at later time points after the index event; a comprehensive stratification may prevent the re-occurrence of TTC through the aid of heart failure therapeutics or other types of drugs still under investigation. At the 3- and 6-month follow-ups, our subject was asymptomatic from the cardiovascular point of view; however, the longer follow-up of the patient is warranted to obtain more accurate knowledge of the condition.

In contrast to other cases of TTC presented in the literature where, in general, a large inadvertent catecholamine dose led to iatrogenic TTC [24], the dose of epinephrine administered to the patient in the present study was within the recommended doses, although most probably this was additive to the endogenous secretion of catecholamines. Despite the fact that psychiatric conditions are frequently associated with TTC, of note, as demonstrated by a previous systematic review [6], very few of the patients were further on addressed to a psychiatrist or psychologist, as stress cardiomyopathy is merely treated by cardiologists, thus excluding the psychiatric dimension.

Compared to other cases, digoxin toxicity [25] may have played a secondary role in the development of TTC in the case described herein, as the plasmatic concentrations did not reach toxic levels; however, even if digoxin is not the main inducer of toxicity in our patient, in corroboration with other elements, such as epinephrine administration and the psychiatric condition, it may have influenced TTC development.

The occurrence of stress cardiomyopathy in the patient described in this case report is presumably the result of cumulative factors, such as emotional distress and the exogenous administration of epinephrine; however, it can be argued that the patient was at a distance from the emotional event and, consequently, TTC may be more associated with the toxic effects of the metoprolol and catecholamine infusion. Epinephrine in large, supra-therapeutic doses has been shown to be associated with negative effects on cardiovascular functions, such as significantly lower values of cardiac inotropy, cardiac output and mean arterial pressure [26] and, consequently, in some cases with TTC [27]. Compared with epinephrine that has a half-life of only 3 to 5 min and, consequently, has rapid plasmatic clearance, metoprolol succinate is a β1-blocker, with moderate lipophilicity and low membrane stabilizing activity with a prolonged half-life of 3 to 7 days. At the time the patient developed TTC, a significant diminution of the β-blocker effect would have occurred, leaving the receptors prone to the negative effects of catecholamines. Several mechanisms are incriminated in the occurrence of TTC with β-blocker poisoning, such as metabolic disruptions, direct myocardial damage, catecholamine cardiotoxicity or neurological impairment [3]. The intimate pathophysiological mechanisms of TTC are still under research; however, β-blockers, benzodiazepines and digoxin toxicity, along with catecholamines, appear to be closely associated with this acute, unexpected type of cardiomyopathy.

Although, there are case reports of exogenous and endogenous catecholamine-triggered TTC [7,27], the case described in the present study depicts multiple contributors to the development of this disease, such as digoxin and benzodiazepine toxicity, the psychiatric condition of the patient (depression), the cumulative effects of stress and treatment with epinephrine. The published cases and literature reviews refer to either digoxin toxicity [25], psychiatric disorders [6], epinephrine release or administration [7] but do not describe a case where all of these are encountered, raising a red flag on the precautious use of epinephrine as treatment of hemodynamic instability, mainly in situations when multiple contributors can lead to stress cardiomyopathy.

## 4. Conclusions

The present study describes the case of a patient with stress cardiomyopathy. This occurred in a female subject who attempted suicide, poisoning herself with β-blockers, benzodiazepines and digoxin. The mechanisms inducing TTC are presumed to be cumulative: a combination of β-blockers, digoxin and benzodiazepine toxicity, and increased levels of exogenous and endogenous stress-related catecholamines. Paramount drugs for the treatment of cardiovascular instability, epinephrine and other similar substances should be carefully administered to subjects with multi-drug poisoning, as these may have the potential of inducing stress cardiomyopathy.

## Figures and Tables

**Table 1:** Blood analysis results of the patient upon presentation in the emergency department.

Parameter	Value	Normal Range
Leucocytes	6500/mm^3^	4000–10,000/mm^3^
Hemoglobin	13.2 g/dL	11–17.3 g/dL
Platelets	324,000/mm^3^	150–472,000 mm^3^
Sodium	138 mmol/L	133–146 mmol/L
Potassium	4.6 mmol/L	3.5–5.3 mmol/L
Urea	6 mmol/L	2.5–7.8 mmol/L
Creatinine	0.9 mg/dL	0.8–1.1 mg/dL
Glycemia	87 mg/dL	70–99 mg/dL
Alanine aminotransferase	12 IU/L	0–35 IU/L
Aspartate aminotransferase	21 IU/L	8–33 IU/L
Thyroid-stimulating hormone	1.3 µIU/L	0.44–5 µIU/L
Lactate	1.9 mmol/L	0.5–1 mmol/L
Digoxin	4.8 ng/ml	0.6–2.4 ng/mL
Metoprolol	1 mg/L	0.1–0.14 mg/L
Benzodiazepines	1800 ng/ml	2–1000 ng/mL
Acetaminophen	61 µg/mL	60–130 µg/mL
Oxycodone	20 ng/mL	10–40 ng/mL
Phenobarbital	38 µg/mL	20–80 µg/mL

## Data Availability

Upon request from the correspondent author.
